# Genome-wide analysis of DNA methylation and risk of cardiovascular disease in a Chinese population

**DOI:** 10.1186/s12872-021-02001-w

**Published:** 2021-05-12

**Authors:** Yan Gao, Huifang Pang, Bowang Chen, ChaoQun Wu, Yanping Wang, Libo Hou, Siming Wang, Dianjianyi Sun, Xin Zheng

**Affiliations:** 1 National Clinical Research Center for Cardiovascular Diseases, State Key Laboratory of Cardiovascular Disease, Chinese Academy of Medical Sciences and Peking Union Medical College, Fuwai Hospital, National Center for Cardiovascular Diseases, 167 Beilishi Road, Beijing, 100037 People’s Republic of China; 2 Department of Epidemiology and Biostatistics, School of Public Health, Peking University Health Science Center, Beijing, People’s Republic of China

**Keywords:** EWAS, DNA methylation, CVD

## Abstract

**Background:**

Systemic studies of association of genome-wide DNA methylated sites with cardiovascular disease (CVD) in prospective cohorts are lacking. Our aim was to identify DNA methylation sites associated with the risk of CVD and further investigate their potential predictive value in CVD development for high-risk subjects.

**Methods:**

We performed an epigenome-wide association study (EWAS) to identify CpGs related to CVD development in a Chinese population.We adopted a nested case–control design based on data from China PEACE Million Persons Project. A total of 83 cases who developed CVD events during follow-up and 83 controls who were matched with cases by age, sex, BMI, ethnicity, medications treatment and behavior risk factors were included in the discovery stage. Genome-wide DNA methylation from whole blood was detected using Infinium Human Methylation EPIC Beadchip (850 K). For significant CpGs [FDR(false discovery rate) < 0.005], we further validated in an independent cohort including 38 cases and 38 controls.

**Results:**

In discovery set, we identified 8 significant CpGs (FDR < 0.005) associated with the risk of CVD after adjustment for cell components, demographic and cardiac risk factors and the first 5 principal components. Two of these identified CpGs (cg06901278 and cg09306458 in *UACA*) were replicated in another independent set (*p* < 0.05). Enrichment analysis in 787 individual genes from 1036 CpGs in discovery set revealed a significant enrichment for anatomical structure homeostasis as well as regulation of vesicle-mediated transport. Receiver operating characteristic (ROC) analysis showed that the model combined 8 CVD-related CpGs with baseline characteristics showed much better predictive effect for CVD occurrence compared with the model with baseline characteristics only [AUC (area under the curve) = 0.967, 95% CI (0.942 − 0.991); AUC = 0.621, 95% CI (0.536 − 0.706); *p* = 9.716E-15].

**Conclusions:**

Our study identified the novel CpGs associated with CVD development and revealed their additional predictive power in the risk of CVD for high-risk subjects.

**Supplementary information:**

The online version contains supplementary material available at 10.1186/s12872-021-02001-w.

## Introduction

Cardiovascular disease (CVD), a complex disease that is attributed to the interaction between environmental and genetic factors, is the leading cause of death in most countries [1, 2]. It is necessary to elucidate the underlying biological mechanisms of CVD to enable early diagnosis and treatment of the disease.

For the development of CVD, it is widely accepted that age, sex, high blood pressure, smoking, dyslipidemia, and diabetes are the major risk factors. Furthermore, over the past few years, many genome-wide association studies (GWAS) have identified relevant genetic polymorphisms as risk factors, but the results that known risk loci only account for a small proportion of risk are not encouraging for personal risk prediction based on genotyping [3]. This suggests other risk factors possibly affect the development of CVD between genetics and environmental factors, such as epigenetics.

Epigenetics is based on stable and heritable alterations in gene expression without changes in the DNA sequence itself [4]. DNA methylation is a crucial type of epigenetic modification, by which a methyl group covalent bonds to the C5 position of cytosine in 5′-C-phosphate-G-3′ (CpG) dinucleotides, ultimately regulating the gene transcription activity and alter biological functions [5]. Previous studies have demonstrated associations of DNA methylation with atherosclerosis [6, 7], ischemic heart disease [8], coronary heart disease (CHD) [8], coronary artery disease [9] and acute coronary syndrome (ACS) [10]. Besides, DNA methylation is suggested as a biomarker for CVD risk [11]. However, the previous evidences were based on cross-sectional studies, lack of the rationale of temperal causality. Systemic identification of genome-wide DNA differential CpGs and analysis of their association with CVD risk are lacking.

In the present study, our main objective is to identify CpGs associated with the risk of CVD and further investigate their potential predictive value in CVD development for high-risk subjects. We first examined the association of genome-wide DNA methylation patterns from whole blood samples in 83 pairs of case–control participants at high-risk of CVD using Infinium Human Methylation EPIC Beadchip (850 K). For identified significant CpGs, we further validated their association with the development of CVD in another independent cohort. And enrichment analysis in Gene Ontology (GO) terms was conducted to identify significantly enriched categories. Finally, we performed the ROC analysis to assess the predictive value of genome-wide significant CpGs for the risk of CVD.

## Methods

### Study design and study samples

The study samples were collected from the China PEACE Million Persons Project (MPP) cohort. The design and conduct of the project pilot have been previously described in detail [12]. In brief, the China-PEACE MPP is a national, population-based screening project funded by the Chinese government to identify individuals at high-risk of CVD. The project enrolled community population if they were 35–75 years old and could confirm their residence in a selected region including 141 primary health care sites (88 rural counties and 53 urban districts) from all 31 provinces in the mainland of China from September 15, 2014 to June 20, 2017. The participants were initially screened for high-risk of CVD by measurements of blood pressure, height, weight and blood lipid, and a questionnaire on cardiovascular-related health status. The subjects at high-risk of CVD were identified if they met at least one of four criteria: 1. Medical history of myocardial infarction (MI), percutaneous coronary intervention (PCI), coronary artery bypass grafting (CABG) treatment, or stroke; 2. High blood pressure defined as systolic blood pressure (SBP) ≥ 160 mmHg or diastolic blood pressure (DBP) ≥ 100 mmHg; 3. Dyslipidemia defined as low-density lipoprotein cholesterol (LDL-C) ≥ 160 mg/dL (4.14 mmol/L) or high-density lipoprotein cholesterol (HDL-C) < 30 mg/dL (0.78 mmol/L); 4. Risk of CVD in 10 years ≥ 20% based on WHO/ISH Cardiovascular Risk Prediction Charts for the Western Pacific Region B [13]. The high-risk participants received further health assessments, including electrocardiography, ultrasound scan, blood and urine analysis, and a questionnaire on lifestyle and medical history and were followed up every year to collect information on medication adherence, risk factor control, and any hospitalization. The data were collected from standardized in-person interviews by trained medical staff. The central ethics committee at the China National Center for Cardiovascular Disease (NCCD) approved this project. All participants had completed a written informed consent before participation in the project.

In the present study, we performed a nested case–control study based on the high-risk participants from the MPP cohort to examine and validate the association of genome-wide CpGs with the development of CVD. The inclusion criteria: 1. Blood samples were collected after January 1, 2016; 2. Two years follow-up data was available; 3. The questionnaire for the high-risk participants was available. Patients were excluded if they have self-reported any of medical history of cancer, chronic liver and kidney disease, infectious diseases, or any CVD at baseline.

Among the above eligible population, case (subjects who developed cardiovascular events) and control (subjects who did not develop cardiovascular events) were 1:1 matched for age (within 1 year), sex, BMI, the month of blood collection (within 1 month), medications treatment (antiplatelet medications and statins), ethnicity (han or non-han), current smoking status, and current alcohol intake at baseline. Then, 121 pairs were randomly sampled from the above eligible case–control pairs. Among them, 83 pairs case–control and 38 pairs case–control were randomly selected and regarded as the discovery set and the replicated set, respectively. Additional file shows this in more detail. [see Additional file 2: Figure S1].

### Clinical variables

Cardiovascular events were self-reported by participants during follow-up visits. In the present study, hypertension was defined as SBP ≥ 140 mmHg, or DBP ≥ 90 mmHg, or self-reported any antihypertensive drugs. Hyperlipidemia was defined as total cholesterol (TC) ≥ 6.2 mmol/L or LDL-C ≥ 4.1 mmol/L according to Chinese guideline for adult’s blood lipid, or self-reported lipid-lowering drug. Diabetes was defined as blood glucose greater than 7 mmol/L after at least 8 h after last meal or random blood glucose greater than 11.1 mmol/L, or any kind of hypoglycemic agents. Details of these variables are shown in Table 1.

**Table 1:** Participant characteristics

Variables	Discovery set			Replication set		
	Cases (n = 83)	Controls (n = 83)	*p*	Cases (n = 38)	Controls (n = 38)	*p*
Basic characteristic						
Age	62 (8)	62 (8)	0.986	61 (9)	61 (9)	1
Male	47 (56.6)	47 (56.6)	1	20 (52.6)	20 (52.6)	1
Ethnicity (han)	78 (94.0)	78 (94.0)	1	37 (97.4)	37 (97.4)	1
Current smoker	26 (31.3)	26 (31.3)	1	11 (28.9)	11(28.9)	1
Smoking index^a^	820 (490, 940)	545 (305, 860)	0.180	570 (320, 860)	620 (480, 880)	0.804
Drinker	11 (13.3)	11 (13.3)	1	4 (10.5)	4 (10.5)	1
BMI	25.5 (4.0)	25.5 (4.0)	0.956	26.0 (3.5)	26.0 (3.5)	0.983
Diagnosis						
Hyperlipidemia	19 (22.9)	9 (10.8)	**0.038**	5 (13.2)	6 (15.8)	0.744
Hypertension	79 (95.2)	76 (91.6)	0.349	34 (89.5)	33 (86.8)	0.723
Diabetes	32 (38.6)	31 (37.3)	0.873	12 (31.6)	16 (42.1)	0.342
Family history of CHD	6 (7.2)	3 (3.6)	0.304	0 (0.0)	2 (5.3)	0.152
Clinical exams						
Systolic pressure	164 (21)	163 (20)	0.553	165 (22)	158 (21)	0.137
Diastolic pressure	95 (13)	91 (12)	**0.019**	96 (13)	91 (13)	0.150
HbA1c (%)	4.9 (4.6, 5.3)	5.0 (4.6, 5.4)	0.639	4.8 (4.6, 5.3)	4.9 (4.7, 5.2)	0.714
hsCRP (mg/L)	0.8 (0.5, 2.1)	1.1 (0.5, 2.4)	0.317	1.2 (0.7, 2.4)	1.1 (0.6, 2.3)	0.373
HDL-C (mmol/L)	0.8 (0.7, 1.0)	0.8 (0.7, 1.1)	0.981	0.9 (0.7, 1.1)	0.9 (0.7, 1.0)	0.593
LDL-C(mmol/L)	2.6 (2.1, 3.2)	2.4 (2.0, 3.0)	0.188	2.6 (2.1, 3.0)	2.6 (2.2, 3.3)	0.461
TG (mmol/L)	1.3 (0.9, 1.9)	1.1 (0.8, 1.6)	0.223	1.3 (1.0, 1.7)	1.5 (0.9, 2.0)	0.262
TC (mmol/L)	4.3 (3.6, 5.0)	4.2 (3.5, 5.1)	0.549	4.2 (3.7, 5.0)	4.4 (3.7, 5.5)	0.424
Events during follow up						
CVD death^b^	18 (21.7)	0	NA	5 (13.2)	0	NA
CVD admission^c^	65 (78.3)	0	NA	33 (86.8)	0	NA
AMI	24 (28.9)	0	NA	4 (10.5)	0	NA
Ischemic stroke	41 (49.4)	0	NA	29 (76.3)	0	NA

^a^The smoking index was calculated based on current smokers, Smoking index = the number of cigarettes smoked per day * years of smoking

^b^CVD death was defined as death due to MI, angina, heart failure, ischemic or hemorrhagic stroke

^c^CVD admission is defined as any hospitalization for MI, ischemic stroke, CABG, PCI, and thrombolysis treatment for acute MI or stroke. CHD, coronary heart disease; BMI, body mass index; AMI, acute myocardial infarction; CVD, cardiovascular disease; HbA1c, hemoglobin A1c; hsCRP, high sensitivity C reactive protein; HDL-C, high density lipoprotein cholesterol; LDL-C, low density lipoprotein cholesterol; TG, triglyceride; TC, total cholesterol

In this study, TC, LDL-C, HDL-C, triglycerides (TG), and high-sensitivity C-reactive protein were measured by standardized enzymatic methods using Beckman Coulter AU680 analyzers (Beckman AU reagent). Hemoglobin A1c (HbA1c) was measured by high-performance liquid chromatography on the Arkray ADAMS-A1C HA-8180 analyzer. All tests were completed at the NCCD laboratory.

### Clinical outcomes

CVD events included CVD admission and CVD death. CVD admission was defined as any hospitalization for MI, ischemic stroke, CABG, PCI, and thrombolysis treatment for acute MI or stroke. CVD death was defined as death due to MI, angina, heart failure (HF), ischemic or hemorrhagic stroke reported by patient’s relatives or doctors. For the patients who have developed multiple cardiovascular events, only the earliest one was used in subsequent analyses.

### DNA methylation analysis

Genomic DNA was isolated from human whole blood samples using Chemagic 360 and chemagic DNA Buffy Coat 200 Kit according to the manufacturer’s protocol. The quality and quantity of the extracted genomic DNA was analyzed with a DropSense 96 Spectrophotometer. The samples (500 ng genomic DNA) were treated for sodium bisulfite conversion using the EZ DNA methylation Gold Kit (Zymo Research, USA). And Genome-wide DNA methylation profiles were assessed using the Infinium Human Methylation EPIC BeadChip (850 K) (Illumina, USA) following manufacturers’ protocol, and DNA methylation level was quantified as a β value. β value represents the ratio of the fluorescent signal intensity measured by methylated and unmethylated probes and range from 0 (all copies of the CpG site in the sample are unmethylated) to 1 (all copies of the CpG site in the sample are methylated) [14].

Data quality control was performed using the chip analysis methylation pipeline (ChAMP) package [15] implemented in *R* both in discovery set and in replication set. Probes meeting one of following criteria were removed: (1) probes with detection *p* > 0.01; (2) probes with < 3 beads in at least 5% of samples; (3) all non-CpG probes contained in dataset; (4) all multi-hit probes; (5) probes containing single nucleotide polymorphisms [16]; and (6) all probes located on chromosome X and Y. After quality control, 769,031 CpGs were retained for analysis. The filtered data were normalized by Beta Mixture Quantile dilation (BMIQ) [17]. Additionally, batch effect was adjusted to reduce technical biases by COMBAT, which was evident in a singular value decomposition (SVD) analysis [18].

### Statistical analysis

Differences in baseline characteristics between case and control groups were evaluated by Kruskal–Wallis test for continuous variables or Chi-Square test for categorical variables. Before genome-wide DNA methylation association analysis, we performed principal component analysis (PCA) using 769,031 CpGs in both discovery and replication set, and the first 5 principal components (PCs) with proportion of variance > 1% were included as covariates in model for minimizing the potential technical bias. To investigate the association of CVD outcomes and DNA methylation level at each CpG, we fitted linear regression models using CpGassoc package in *R*, with β as dependent variables and CVD outcomes as independent variables. The models were adjusted for covariates, including age, sex, BMI, current smoking status, hypertension, diabetes mellitus, hyperlipidemia, cell components and the first 5 PCs. Cell components were estimated by the proportions of whole blood (CD4 + T cells, CD8 + T cells, B cells, NK cells, monocytes, and neutrophile granulocyte) using the minfi [19] of *R* package based on Houseman approach. We used the FDR < 0.005 to identify significant CVD-associated CpGs in discovery set.

In replication set, the significant CpGs identified in discovery set were validated by linear regression models using CpGassoc package in *R*. The models were adjusted for the same factors as that in discovery set. The CpGs with *p* < 0.05 in replication set and the consistent direction of effect in both discovery and replication sets were considered as significant replication CpGs.

For significant CpGs, we used the annotation provided by Illumina and University of California Santa Cruz UCSC database (GRCh37/hg19). The genes annotated to CpGs with *p* < 10^–4^ in discovery set were selected for the enrichment analysis. The enrichment analysis in GO terms were conducted with R package 'clusterProfiler', and FDR < 0.05 was used to identify significantly enriched categories.

Furthermore, ROC analysis was performed to assess the predictive value of significant CpGs in CVD outcomes. There were two prediction models included in ROC analysis: 1) model with baseline characteristics only; 2) model with both baseline characteristics and the significant CpGs. As a reference model, baseline characteristics included age, gender, BMI, current smoking status, current alcohol intake, hypertension, diabetes mellitus, hyperlipidemia, antiplatelet medication, stains treatment and family history of coronary heart disease. Statistical analysis were performed using the SAS version 9.4 and R version 3.5.

## Results

### Characteristics of the study subjects

The baseline characteristics of the study subjects are shown in Table 1. In discovery set, there were 83 CVD cases (65 admissions for CVD, 18 died of CVD) and 83 controls with mean age of 62 years. The replication set included 38 CVD cases (33 admissions for CVD, 5 died of CVD) and 38 controls with mean age of 61 years. As the study subjects were from the high-risk participants of the MPP cohort, the proportion of hypertension subjects in our study cohort was approximately as high as 90%. There was no significant difference in most variables between the case and control group in two cohorts, except for hyperlipidemia (*p* = 0.038) and diastolic pressure (*p* = 0.019) in the discovery set.

### Epigenome-wide association analysis

**Fig. 1:**
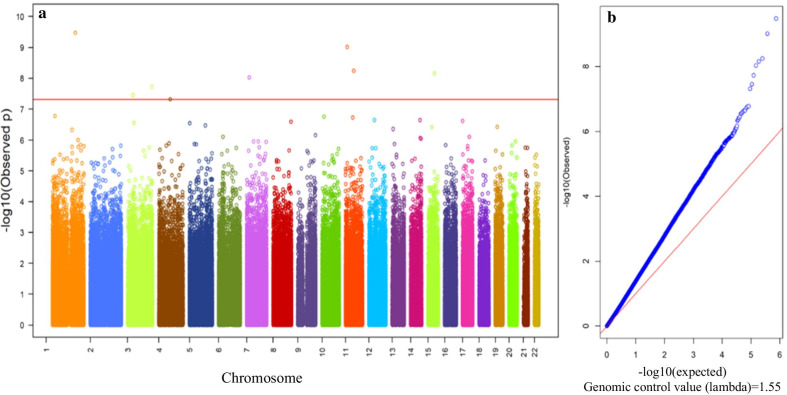
Manhattan plot (**a**) and QQ plot (**b**) in the discovery set. In the Manhattan plot, red line represents -log10 *P* at false discovery rate (FDR) = 0.005

Before performing a epigenome-wide association analysis, we did SVD analysis and PCA based on plate number, chip number and date of test for examining batch effect in the normalized data by BMIQ and the further processed data by COMBAT, respectively. SVA analysis showed that batch effect was almost completely controlled after adjustment in different chips and different sample locations by COMBAT [see Additional file 2: Figure S2, S3]. In the PCA, no significant aggregations were found between different plates, different chips and different date of test (see Additional file 2: Figure S4). After that, PCA based on study samples was conducted using 769,031 CpGs in both discovery and replication set, and the first 5 PCs with proportion of variance > 1% were included as covariates in the genome-wide association analysis by linear regression model [see Additional file 2: Table S1, S2]. The results showed that eight CpGs achieved a genome-wide significance level (FDR < 0.005) after adjustment for age, gender, BMI, current smoking status, hypertension, diabetes mellitus, hyperlipidemia, cell compositions and the first 5 PCs (Fig. 1 and Table 2). Six of 8 CVD-associated CpGs were located in *SPON1* gene body (cg11651314), in *PACS1* gene body (cg03914662), in TSS200 of the *UACA* gene (cg09306458), in *CCDC50* gene body ( cg05946546), in 1stExon of the *CYP8B1* gene ( cg07655795), and in *HSD17B11* gene body ( cg02518222), respectively (Table 2). Of them, increased methylation level at CpG cg07655795 (*CYP8B1*) was associated with an increased risk of CVD, while decreased methylation level at the remaining 7 CpGs were associated with an increased risk of CVD.

**Table 2:** Association analysis for CVD outcomes with genome-wide significant CpGs identified in the discovery set (FDR < 0.005)

CpG	Gene	Relation to gene	Discovery set				Replication set		
			Effect*	SE	*p*	FDR	Effect	SE	*p*
cg06901278	–	–	− 0.022	0.003	3.37E−10	< 0.001	− 0.015	0.006	0.010
cg11651314	*SPON1*	Body	− 0.033	0.005	9.70E−10	< 0.001	− 0.012	0.007	0.072
cg03914662	*PACS1*	Body	− 0.0177	0.003	5.73E−09	0.0013	− 0.004	0.005	0.415
cg09306458	*UACA*	TSS200	− 0.009	0.001	7.01E−09	0.0013	− 0.005	0.002	0.016
cg05359217	*–*	–	− 0.012	0.002	9.34E−09	0.0014	− 0.002	0.003	0.591
cg05946546	*CCDC50*	Body	− 0.017	0.003	1.88E−08	0.0024	− 0.003	0.005	0.538
cg07655795	*CYP8B1*	1stExon	0.024	0.004	3.55E−08	0.0039	0.007	0.006	0.249
cg02518222	*HSD17B11*	Body	− 0.042	0.007	4.85E−08	0.0047	− 0.017	0.011	0.111

TSS, transcription start site; FDR, false discovery rate. *Effect value represents change in β value per change from control to case

For the 8 CVD-associated CpGs (FDR < 0.005) identified in discovery set, we performed replication analysis in an independent cohort (38 cases and 38 controls). Two CpGs of them (cg09306458, cg06901278) were replicated (*p* < 0.05) with consistent direction of effect in both sets (Table 2). In two replicated CpGs, cg09306458 was located in regulatory region of the *UACA* gene, and cg06901278 had no annotation gene.

### Enrichment analysis

**Fig. 2:**
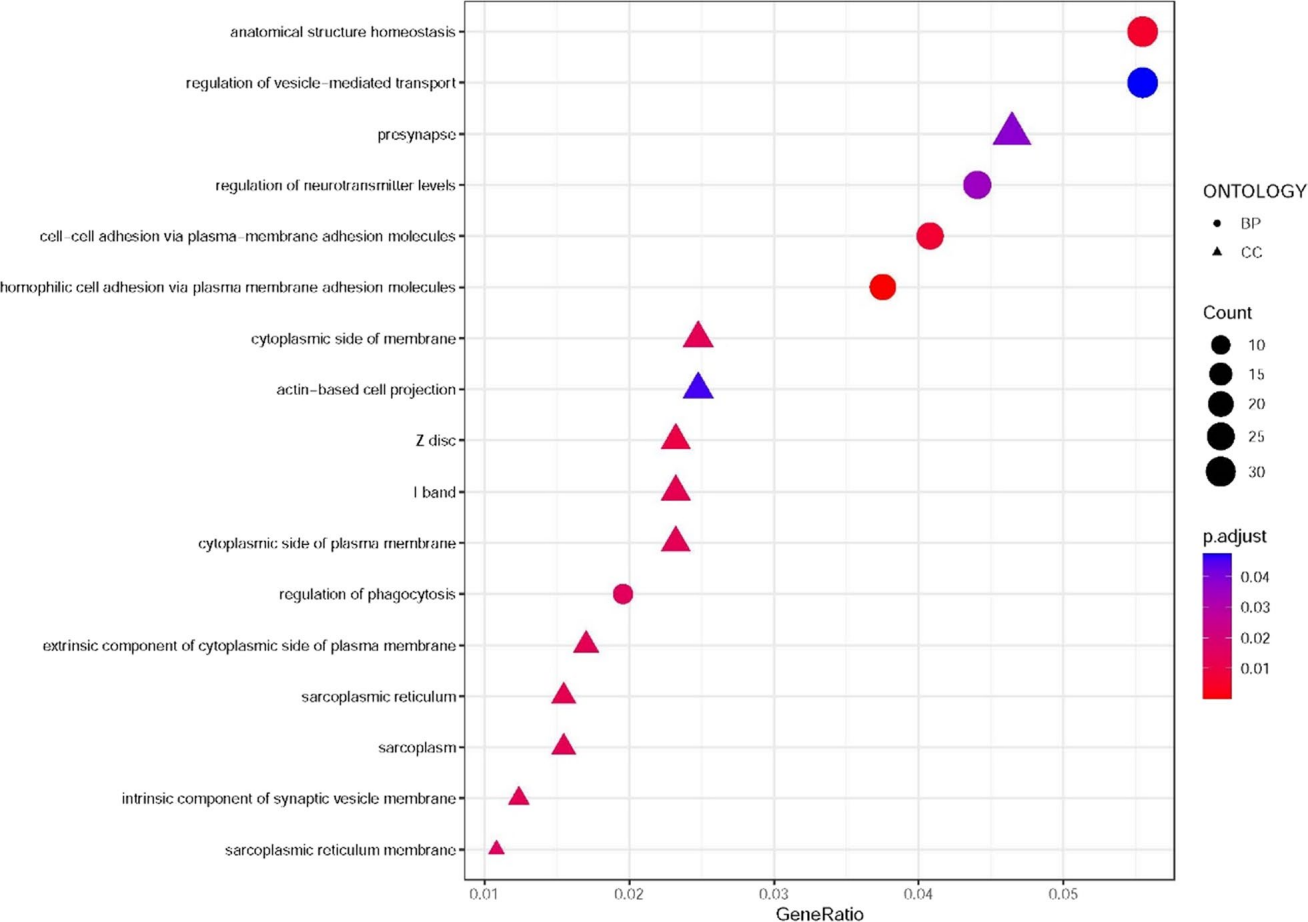
Top categories ranked according to their statistical significance. BP, biological processes. CC, cellular component. GO, Gene Ontology

Go term enrichment analysis were conducted to identify the possible functions affected by annotation genes. A total of 787 individual genes from 1036 CpGs with *p* < 10^–4^ in discovery set were used for enrichment analysis. There were 17 significantly functional categories identified (FDR < 0.05), including 6 categories in biological processes (BP), and 11 categories in cellular component (CC) (Fig. 2) (see Additional file 1: Excel S1). The most enriched categories were significant enrichment for anatomical structure homeostasis as well as regulation of vesicle-mediated transport, which were located in BP group (Fig. 2).

### ROC analysis

**Fig. 3:**
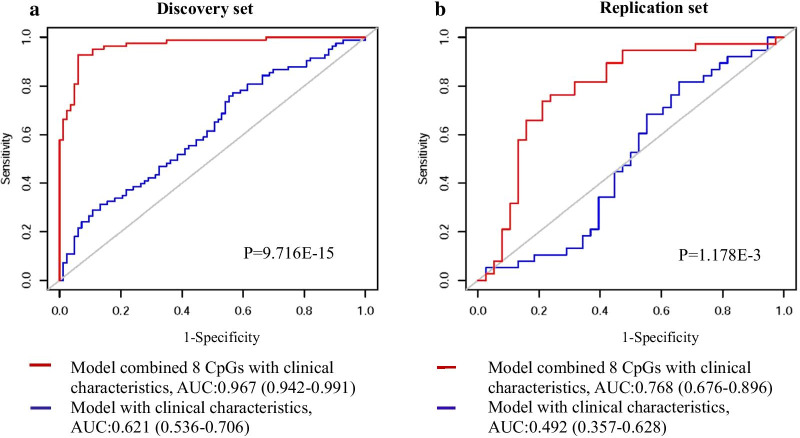
Receiver operating characteristic (ROC) analysis of the sensitivity and specificity of CVD prediction by the model combined CVD-related CpGs and baseline characteristics. **a** indicates the discovery set; **b** indicates the replication set. AUC, area under the curve. The baseline characteristics were assessed as a reference model, including age, gender, BMI, current smoking status, current alcohol intake, hypertension, diabetes mellitus, hyperlipidemia, antiplatelet medication, stains treatment and family history of coronary heart disease

ROC curves were drawn to assess the specificity and sensitivity of CVD prediction on the basis of 8 CVD-related CpGs identified in discovery set and baseline characteristics as a reference model. As shown in Fig. 3a, the model with both 8 CVD-related CpGs and baseline characteristics showed an excellent predictive effect for CVD occurrence and much better than the model with baseline characteristics only (AUC = 0.967, 95% CI (0.942 − 0.991); AUC = 0.621, 95% CI (0.536 − 0.706); *p* = 9.716E-15). In replication set, we also found the similar trend that the model combined 8 CVD-related CpGs and baseline characteristics showed significant predictive capacity compared with the reference model with baseline characteristics only (AUC = 0.786, 95% CI (0.676 − 0.896); AUC = 0.492, 95% CI (0.357 − 0.628); *P* = 1.178E-3) (Fig. 3b).

## Discussion

The present study provides evidence that DNA methylation level in whole blood DNA was associated with the risk of CVD at specific CpGs in high-risk Chinese population. In the discovery analysis, we identified 8 significant CVD-related CpGs after adjustment for conventional covariates and the first 5 PCs. And two of these CVD-associated CpGs were replicated in an independent cohort. Enrichment analysis revealed that the genes annotated to CpGs with *p* < 10^–4^ in discovery set were related with significant enrichment for anatomical structure homeostasis as well as regulation of vesicle-mediated transport. ROC analysis suggested that CVD-associated CpGs identified could provide additional predictive power for CVD occurrence in high-risk subjects.

In this study, there were some strengths in minimizing the potential confounding effect to ensure the findings reliable. First, the case-controls were strictly matched for the traditional risk factors of CVD and factors probably affecting DNA methylation status. Second, normalized methylation data was processed for reducing batch effect by COMBAT, and examined by SVD analysis and PCA. Third, we have adjusted for age, sex, BMI, cell compositions, hypertension, diabetes mellitus, hyperlipidemia, and PCs in regression model. Fourth, our prospective design fits a temporal relationship between the DNA methylation level and CVD outcomes, which elucidated the causal relationship more reasonablly. In addition, Infinium Human Methylation EPIC Beadchip (850 K) almost doubled the number of CpGs on the 450 K array which was widely used in previous studies, which improves coverage within intergenic regions, enhancers, and distal regulatory elements [20]. In view of the above reasons, we believe that our results are reliable although some degree of inflation in the QQ-plot (λ = 1.55) was observed in our study, which might be due to the polygenic effect of DNA methylation on CVD or the residual confounding that was not completely controlled in the linear regression models.

In discovery phase, there were 8 CpGs significantly associated with CVD development in the Chinese population. The CpGs cg09306458 was located in regulatory region of the *UACA* gene, which encodes a protein that contains ankyrin repeats and coiled coil domains and likely plays a role in apoptosis. A study of the Danish National Birth Cohort (608 cases vs 626 control) identified the *UACA* gene at the cg12157761 associated with the offspring of women with gestational diabetes mellitus [21]. In addition, a meta-analysis showed that newborn blood DNA CpG cg01244124 located in *UACA* was negatively associated with sustained maternal smoking during pregnancy [22]. Though the gene *UACA* in responding to different CpGs among the various studies, the results of these two studies were not contradictive with our findings, in that diabetes mellitus and smoking were the classic risk factor of CVD and may induce DNA methylation alteration at specific site to result in the outcome of CVD.

The CpGs cg11651314 located in the gene body region of *SPON1* was also associated with CVD development at genome-wide level in our study. *SPON1* encodes the developmentally regulated protein F-spondin, which has been reported to be a putative ligand for the amyloid precursor protein [23]. We observed decreased methylation at this CpGs, which was associated with the increased risk of CVD, and a previous study for DNA methylation in adipose tissue also showed *SPON1* in corresponding to the site cg23284931 was negatively correlated with type 2 diabetes [24]. Additionally, proteomic profiling study in community-based prospective cohorts revealed that higher levels of circulating protein SPON1 at baseline was associated with worsened systolic function and HF incidence independently of established risk factors [25]. So it is likely that the hypomethylated status could enhance the expression of *SPON1* and contribute to the pathogenesis of CVD. However, in the atherosclerotic human aorta sample, *SPON1* was significantly hypermethylated compared with the plaque-free intima [26], reflecting the difference of DNA methyaltion pattern at certain genes in different tissues.

The hypermethylated CpGs cg07655795 identified in EWAS was located in the 1stExon region of *CYP8B1* (Cytochrome P450 Family 8 Subfamily B Member 1). CYP8B1 was one of the key enzymes of bile acids synthesis, and ablating *CYP8B1* in mice led to reduced absorption of dietary triglyceride with intact triglyceride hydrolysis and improved insulin sensitivity, suggesting CYP8B1 as a potential therapeutic target for obesity and diabetes [27]. Another significant CpG cg07655795 associated with CVD risk in our study was located in body region of gene *HSD17B11* (Hydroxysteroid 17-βDehydrogenase 11). In a integrated microarray analysis, *HSD17B11* was one of the up regulated genes in coronary artery disease, and pathway enrichment analysis revealed that differentially expressed genes in coronary artery disease were mostly enriched in the superpathway of steroid hormone biosynthesis, and so on [28]. In addition, our findings showed that CpGs cg03914662 and cg05946546 were located in the body region of *PACS1* and *CCDC50*, respectively. *PACS1* encodes phosphofurin acidic cluster sorting protein 1, which is a multifunctional membrane traffic regulator that plays an important role in organ homeostasis [29]. *CCDC50* encodes Ymer, an effector of epidermal growth factor (EGF)–mediated cell signaling that is ubiquitously expressed in different organs and has been suggested to inhibit down-regulation of the EGF receptor [30, 31]. Up to date, no evidence has associated DNA methylation with CVD or CVD risk factor in regard to *PACS1* and *CCDC50*. Therefore, it’s essential to further validate the CpG site cg03914662 and cg05946546 in a larger independent cohort and assess the association with the expression of its annotation genes.

There were no overlap between our identified CVD-related CpGs and the sites reported in previous MI-associated [32, 33] and ACS-associated EWAS studies^[34]^with whole blood sample. Rask-Andersen et al. [32] identified 211CpGs mapped to 196 annotation genes in individuals with a history of MI from northern Sweden population, and 42 genes among them had been described to be related to cardiac function, CVD, cardiogenesis and recovery after ischemic episode. Another study identified 3 CpGs cg07786668 in *ZFHX3*, cg17218495 in *SMARCA4* and cg06642177 in *SGK1* that significantly associated with MI in Japan [33]. Li Jun and colleagues [34] identified 47 ACS-associated CpGs annotated to 44 individual genes at both whole blood and cell level in 102 ACS patients and 101 controls from Chinese population. This lack of overlap between our CVD-related CpGs and MI- or ACS-associated CpGs may be due to the following reasons: (i) Our prospective cohort study is different from the cross-sectional cohort in other studies; (ii) Infinium Human Methylation EPIC Beadchip (850 K) has a larger number of CpGs compared with the 450 K array widely used in previous studies; (iii) The CVD outcomes in our study were not exactly the same as the previous studies. However, our ROC analysis revealed that the model combined genome-wide CVD-associated CpGs with baseline characterastics provided significant predictive capacity for CVD development in high-risk population. Therefore, our identified CpGs may serve as potential biomarkers in clinical risk assessment for CVD.

In present study, Go enrichment ananlysis showed that the genes annotated to CpGs with *p* < 10^–4^ in discovery set were involved in anatomical structure homeostasis as well as regulation of vesicle-mediated transport that may suggest a complex impairment of cellular function [32] in the occurrence of CVD. However, none of 6 CVD-related annotated genes identified in discovery set was overlapped with the enriched genes in GO enrichment analysis. In complex diseases (e.g., cardiovascular disease), the contribution of most genes to the occurrence of disease is small, which may result in these genes not easy to be identified by EWAS, especially given the small sample size of this cohort. Therefore, these genes may not have been included in the enrichment analysis, although we have aleady included the genes annotated to CpGs with *p* < 10^–4^. This may be a reason why the results of enrichment analysis did not overlap with the main results of the study. Nevertheless, considering the good prediction effect in ROC analysis, we believe that it is promising to use these CpGs found in the study as potential biomarkers for predicting the occurrence of CVD.

The present study has some limitations. First, the sample size was small both in discovery and replication sets, so we should validate our results in a larger independent cohort. Second, we used whole blood samples to examine the DNA methylation level, which may not directly reflect the status of the target tissue. Third, we did not assess the association between gene expression and CpGs which would improve the confidence of the results. Moreover, self-reported medical history might introduce recall bias, although the data were collected by trained medical staff. Finally, our findings need further validation in other ethnicity groups and population with relatively low CVD risk.

## Conclusion

In conclusion, DNA methylation level at 8 novel CpGs was associated with CVD development in a Chinese population, and two of them were replicated in another independent cohort. Enrichment analysis in discovery set revealed a significant enrichment for anatomical structure homeostasis and regulation of vesicle-mediated transport. ROC analysis showed that the model with both CVD-associated CpGs and baeline characterastics had excellent predictive capacity for CVD occurence in high-risk subjects. Future studies ought to verify the predictive effect of these CpGs on CVD in other ethnicity groups and population with relatively low CVD risk and further elucidate the potential functional mechanism of CVD-related CpGs.

## Supplementary information

**Additional file 1: Figures S1–S4 and Tables S1–S2.** **Figure S1.** Flow chart of study sample selection. **Figure S2.** Singular value decomposition (SVD) analysis by data before processing by COMBAT. **Figure S3.** Singular value decomposition (SVD) analysis by data after processing by COMBAT. **Figure S4.** Principal component analysis (PCA) based on plate number (A), chip number (B), date of test (C) and gender (D). **Table S1.** The top 5 principal components (PCs) with proportion of variance more than 0.01 used for epigenomewide association analysis in the discovery set. **Table S2.** The top 5 principal components (PCs) with proportion of variance more than 0.01 used for epigenomewide association analysis in the replication set.

**Additional file 2: Appendix 1–3. Appendix 1.** Significantly functions categories in Gene Ontology (GO) enrichment analysis in discovery set. **Appendix 2.** Significant Differentially Methylated Regions (DMRs) with annotated genes by hump-hunting method in the discovery group. **Appendix 3.** Significant Differentially Methylated Regions (DMRs) with annotated genes by Probe Lasso method in the discovery group.

## Data Availability

The China PEACE Million Persons Project is a major national program, and as the government policy stipulates, it is not permissible for the researchers to make the raw data publicly available at this time. All data genetated during this study are included in this manuscript and its supplementary information files.

## Abbreviations

CVD: Cardiovascular disease
EWAS: Epigenome-wide association study
FDR: False discovery rate
ROC: Receiver operating characteristic
AUC: Area under the curve
GWAS: Genome-wide association studies
ACS: Acute coronary syndrome
MI: Myocardial infarction,
PCI: Percutaneous coronary intervention
CABG: Coronary artery bypass grafting
SBP: Systolic blood pressure
DBP: Diastolic blood pressure
LDL-C: Low-density lipoprotein cholesterol
HDL-C: High-density lipoprotein cholesterol
NCCD: National Center for Cardiovascular Disease
TC: Total cholesterol
TG: Triglycerides
HF: Heart failure
PCs: Principal components
BMI: Body mass index
CHD: Coronary heart disease
AMI: Acute myocardial infarction
HbA1c: Hemoglobin A1c
hsCRP: High sensitivity C reactive protein
TSS: Transcription start site
UTR: Untraslated region
PCA: Principal component analysis
BMIQ: Beta Mixture Quantile dilation
SVD: Singular value decomposition
CHAMP: Chip analysis methylation pipeline
