# Supplementary material for: Genome-wide analysis of DNA methylation and risk of cardiovascular disease in a Chinese population
Source: BMC Cardiovasc Disord. 2021 May 12;21:240. doi: 10.1186/s12872-021-02001-w (PMC8117656; doi:10.1186/s12872-021-02001-w)
Supplement: Supplementary file 2 — Additional file 2: Appendix 1–3. Appendix 1. Significantly functions categories in Gene Ontology (GO) enrichment analysis in discovery set. Appendix 2. Significant Differentially Methylated Regions (DMRs) with annotated genes by hump-hunting method in the discovery group. Appendix 3. Significant Differentially Methylated Regions (DMRs) with annotated genes by Probe Lasso method in the discovery group. [file 12872_2021_2001_MOESM2_ESM.docx]

Appendix 1. Significantly functions categories in Gene Ontology (GO) enrichment analysis in discovery set

| ONTOLOGY | | Description | | GeneRatio | | BgRatio | | P value |  |
| --- | --- | --- | --- | --- | --- | --- | --- | --- | --- |
| GO:0060249 | BP | | anatomical structure homeostasis | | 0.055 | 0.023 | 2.16E-06 | | |
| GO:0060627 | BP | | regulation of vesicle-mediated transport | | 0.055 | 0.027 | 6.00E-05 | | |
| GO:0098793 | CC | | presynapse | | 0.046 | 0.025 | 6.65E-04 | | |
| GO:0001505 | BP | | regulation of neurotransmitter levels | | 0.044 | 0.019 | 3.70E-05 | | |
| GO:0098742 | BP | | cell-cell adhesion via plasma-membrane adhesion molecules | | 0.041 | 0.015 | 3.91E-06 | | |
| GO:0007156 | BP | | homophilic cell adhesion via plasma membrane adhesion molecules | | 0.038 | 0.009 | 7.45E-09 | | |
| GO:0098562 | CC | | cytoplasmic side of membrane | | 0.025 | 0.009 | 1.82E-04 | | |
| GO:0098858 | CC | | actin-based cell projection | | 0.025 | 0.010 | 8.71E-04 | | |
| GO:0030018 | CC | | Z disc | | 0.023 | 0.006 | 1.69E-05 | | |
| GO:0031674 | CC | | I band | | 0.023 | 0.007 | 4.57E-05 | | |
| GO:0009898 | CC | | cytoplasmic side of plasma membrane | | 0.023 | 0.008 | 1.20E-04 | | |
| GO:0050764 | BP | | regulation of phagocytosis | | 0.020 | 0.004 | 1.21E-05 | | |
| GO:0031234 | CC | | extrinsic component of cytoplasmic side of plasma membrane | | 0.017 | 0.005 | 1.58E-04 | | |
| GO:0016529 | CC | | sarcoplasmic reticulum | | 0.015 | 0.003 | 6.27E-05 | | |
| GO:0016528 | CC | | sarcoplasm | | 0.015 | 0.004 | 1.85E-04 | | |
| GO:0098563 | CC | | intrinsic component of synaptic vesicle membrane | | 0.012 | 0.002 | 1.13E-04 | | |
| GO:0033017 | CC | | sarcoplasmic reticulum membrane | | 0.011 | 0.002 | 2.46E-04 | | |

Appendix2．significant Differentially Methylated Regions (DMRs) with annotated genes by hump-hunting method in the discovery group

| seqnames | start | end | width | area | p.value | SYMBOL |
| --- | --- | --- | --- | --- | --- | --- |
| chr10 | 123688099 | 123688420 | 321 | 2.067 | 0.0037 | GPR26 |
| chr11 | 6258935 | 6260271 | 1336 | 1.110 | 0.0222 | CCKBR |
| chr11 | 130787523 | 130790398 | 2875 | 4.729 | 0.0000 | PPP1R10P1 |
| chr13 | 113422162 | 113423268 | 1106 | 1.189 | 0.0109 | ADPRHL1 |
| chr14 | 73712491 | 73712967 | 476 | 1.767 | 0.0027 | PNMA1 |
| chr15 | 101084980 | 101085710 | 730 | 1.449 | 0.0100 | AC090907.2 |
| chr17 | 7287449 | 7288001 | 552 | 1.300 | 0.0069 | SLC2A4 |
| chr18 | 77623409 | 77624513 | 1104 | 1.510 | 0.0087 | AC123786.1 |
| chr19 | 51891210 | 51891559 | 349 | 1.101 | 0.0159 | ZNF649 |
| chr2 | 731073 | 731561 | 488 | 1.290 | 0.0158 | AC092159.1 |
| chr2 | 200467056 | 200469026 | 1970 | 2.887 | 0.0007 | SPATS2L |
| chr4 | 682831 | 683106 | 275 | 1.003 | 0.0243 | SLC49A3 |
| chr4 | 39639007 | 39639380 | 373 | 1.089 | 0.0076 | SMIM14,AC108471.2 |
| chr5 | 1080273 | 1082123 | 1850 | 1.267 | 0.0020 | SLC12A7 |
| chr5 | 23507030 | 23507656 | 626 | 1.224 | 0.0181 | PRDM9 |
| chr5 | 42423615 | 42424075 | 460 | 1.185 | 0.0175 | GHR |
| chr5 | 178986131 | 178986625 | 494 | 1.358 | 0.0134 | GRM6 |
| chr6 | 291687 | 293285 | 1598 | 6.607 | 0.0000 | DUSP22 |
| chr6 | 31148332 | 31148666 | 334 | 3.038 | 0.0011 | CCHCR1 |
| chr6 | 31275551 | 31275881 | 330 | 5.922 | 0.0000 | USP8P1 |
| chr6 | 38682982 | 38683221 | 239 | 1.338 | 0.0061 | GLO1 |
| chr7 | 967834 | 968577 | 743 | 1.161 | 0.0029 | COX19 |
| chr7 | 29186501 | 29187019 | 518 | 1.058 | 0.0192 | CPVL |

Appendix 3. significant Differentially Methylated Regions (DMRs) with annotated genes by Probe Lasso method in the discovery group

| dmrChrom | dmrStart | dmrEnd | dmrSize | dmrP | geneSymbol |
| --- | --- | --- | --- | --- | --- |
| 6 | 29974693 | 29974947 | 255 | 0.0000 | HLA-J; NCRNA00171 |
| 6 | 31103314 | 31112250 | 8937 | 0.0000 | PSORS1C1; PSORS1C2;CCHCR1 |
| 6 | 33245659 | 33245800 | 142 | 0.0000 | B3GALT4 |
| 22 | 21985586 | 21988793 | 3208 | 0.0000 | YDJC;CCDC116 |
| 12 | 89744463 | 89744754 | 292 | 0.0000 | DUSP6 |
| 3 | 196064717 | 196065494 | 778 | 0.0000 | TM4SF19 |
| 6 | 30038571 | 30039669 | 1099 | 0.0000 | RNF39 |
| 16 | 14402147 | 14404682 | 2536 | 0.0001 | MIR365-1 |
| 6 | 32120800 | 32121711 | 912 | 0.0002 | PPT2;PRRT1 |
| 17 | 73314257 | 73318357 | 4101 | 0.0002 | GRB2 |
| 8 | 19539765 | 19540654 | 890 | 0.0003 | CSGALNACT1 |
| 6 | 30459445 | 30461515 | 2071 | 0.0007 | HLA-E |
| 3 | 52529079 | 52529968 | 890 | 0.0009 | STAB1 |
| 7 | 92235353 | 92240386 | 5034 | 0.0010 | CDK6 |
| 11 | 66638863 | 66640022 | 1160 | 0.0012 | PC |
| 12 | 58173362 | 58178252 | 4891 | 0.0014 | FAM119B;TSFM |
| 17 | 41277151 | 41277505 | 355 | 0.0015 | BRCA1;NBR2 |
| 6 | 31644606 | 31648742 | 4137 | 0.0015 | LY6G5C |
| 1 | 2002085 | 2003682 | 1598 | 0.0016 | PRKCZ |
| 7 | 27137009 | 27141937 | 4929 | 0.0016 | HOXA2 |
| 8 | 667868 | 669505 | 1638 | 0.0016 | ERICH1 |
| 6 | 29945000 | 29945178 | 179 | 0.0018 | HCG9 |
| 19 | 3961491 | 3961824 | 334 | 0.0024 | MIR637;DAPK3 |
| 1 | 34631462 | 34634669 | 3208 | 0.0025 | CSMD2;C1orf94 |
| 11 | 70279561 | 70284523 | 4963 | 0.0029 | CTTN |
| 17 | 3713720 | 3718301 | 4582 | 0.0036 | C17orf85 |
| 6 | 33130946 | 33137377 | 6432 | 0.0036 | COL11A2 |
| 6 | 33231060 | 33237787 | 6728 | 0.0042 | VPS52 |
| 16 | 4713998 | 4714887 | 890 | 0.0046 | MGRN1 |
| 3 | 111717580 | 111718523 | 944 | 0.0048 | TAGLN3 |
| 5 | 1814019 | 1818115 | 4097 | 0.0057 | NDUFS6 |
| 17 | 19289908 | 19290797 | 890 | 0.0068 | MFAP4 |
| 3 | 158390065 | 158391265 | 1201 | 0.0068 | LXN;GFM1 |
| 1 | 101704099 | 101706035 | 1937 | 0.0077 | S1PR1 |
| 10 | 126330735 | 126331682 | 948 | 0.0078 | FAM53B |
| 15 | 78628684 | 78632167 | 3484 | 0.0079 | CRABP1 |
| 11 | 63827901 | 63828790 | 890 | 0.0081 | MACROD1 |
| 2 | 175259664 | 175261230 | 1567 | 0.0082 | CIR1;SCRN3 |
| 2 | 219266835 | 219268432 | 1598 | 0.0082 | CTDSP1;MIR26B |
| 5 | 180071606 | 180073218 | 1613 | 0.0083 | FLT4 |
| 7 | 2762657 | 2772432 | 9776 | 0.0083 | GNA12 |
| 16 | 89293071 | 89297116 | 4046 | 0.0084 | ZNF778 |
| 4 | 718010 | 722473 | 4464 | 0.0084 | PCGF3 |
| 6 | 32093930 | 32096559 | 2630 | 0.0084 | ATF6B;FKBPL |
| 11 | 368504 | 369065 | 562 | 0.0091 | B4GALNT4 |
| 12 | 133299988 | 133303471 | 3484 | 0.0091 | ANKLE2 |
| 19 | 35628502 | 35631958 | 3457 | 0.0091 | FXYD1 |
| 6 | 30610247 | 30611854 | 1608 | 0.0091 | C6orf134 |
| 2 | 33700731 | 33701617 | 887 | 0.0106 | RASGRP3 |
| 18 | 13610955 | 13611933 | 979 | 0.0108 | C18orf1 |
| 15 | 49170164 | 49170323 | 160 | 0.0112 | SHC4;EID1 |
| 3 | 11595845 | 11601838 | 5994 | 0.0119 | ATG7;VGLL4 |
| 13 | 113706984 | 113709087 | 2104 | 0.0120 | MCF2L |
| 17 | 27048857 | 27050454 | 1598 | 0.0120 | RPL23A;SNORD4A; SNORD42A;SNORD4B |
| 20 | 55841862 | 55841987 | 126 | 0.0120 | BMP7 |
| 6 | 30854125 | 30857724 | 3600 | 0.0120 | DDR1 |
| 6 | 31845082 | 31856576 | 11495 | 0.0120 | SLC44A4;EHMT2 |
| 14 | 23938551 | 23939601 | 1051 | 0.0122 | NGDN |
| 1 | 228394405 | 228397701 | 3297 | 0.0124 | OBSCN |
| 6 | 170570920 | 170571809 | 890 | 0.0128 | LOC154449 |
| 11 | 2918890 | 2923559 | 4670 | 0.0128 | SLC22A18AS;SLC22A18 |
| 6 | 32164130 | 32164733 | 604 | 0.0129 | GPSM3;NOTCH4 |
| 7 | 23749588 | 23750473 | 886 | 0.0129 | STK31 |
| 6 | 16303157 | 16307202 | 4046 | 0.0149 | ATXN1 |
| 14 | 21944707 | 21945037 | 331 | 0.0149 | RAB2B;TOX4 |
| 7 | 157366134 | 157367777 | 1644 | 0.0154 | PTPRN2;MIR153-2 |
| 19 | 45446657 | 45450702 | 4046 | 0.0170 | APOC2;APOC4 |
| 11 | 118134356 | 118135245 | 890 | 0.0180 | MPZL2 |
| 17 | 33914113 | 33914890 | 778 | 0.0180 | AP2B1 |
| 7 | 96654616 | 96658764 | 4149 | 0.0180 | DLX5 |
| 3 | 182970757 | 182971072 | 316 | 0.0187 | MCF2L2;B3GNT5 |
| 6 | 32048378 | 32053534 | 5157 | 0.0191 | TNXB |
| 17 | 94931 | 97594 | 2664 | 0.0191 | RPH3AL |
| 4 | 13538494 | 13543185 | 4692 | 0.0209 | NKX3-2 |
| 6 | 30680794 | 30682626 | 1833 | 0.0209 | MDC1 |
| 6 | 31511900 | 31515846 | 3947 | 0.0211 | ATP6V1G2;NFKBIL1 |
| 19 | 4768701 | 4770120 | 1420 | 0.0218 | C19orf30;MIR7-3 |
| 17 | 6734551 | 6735328 | 778 | 0.0227 | TEKT1 |
| 6 | 31691208 | 31691957 | 750 | 0.0227 | C6orf25 |
| 10 | 681348 | 683669 | 2322 | 0.0227 | DIP2C |
| 16 | 57830594 | 57834363 | 3770 | 0.0248 | KIFC3 |
| 4 | 3370437 | 3373070 | 2634 | 0.0251 | RGS12 |
| 16 | 90113448 | 90114617 | 1170 | 0.0290 | LOC100130015 |
| 2 | 236720238 | 236722270 | 2033 | 0.0290 | AGAP1 |
| 6 | 5142055 | 5146100 | 4046 | 0.0290 | LYRM4 |
| 7 | 130353861 | 130354218 | 358 | 0.0290 | TSGA13;COPG2 |
| 7 | 87935689 | 87936466 | 778 | 0.0292 | STEAP4 |
| 7 | 97920005 | 97924781 | 4777 | 0.0316 | BAIAP2L1;BRI3 |
| 10 | 132890601 | 132892088 | 1488 | 0.0321 | TCERG1L |
| 19 | 10792398 | 10793435 | 1038 | 0.0321 | ILF3 |
| 6 | 30069974 | 30071648 | 1675 | 0.0321 | TRIM31 |
| 7 | 65418767 | 65421691 | 2925 | 0.0346 | VKORC1L1 |
| 22 | 32341232 | 32341637 | 406 | 0.0353 | C22orf24;YWHAH |
| 6 | 29637559 | 29641815 | 4257 | 0.0353 | MOG;ZFP57 |
| 5 | 433017 | 437289 | 4273 | 0.0358 | AHRR |
| 6 | 31600304 | 31604333 | 4030 | 0.0361 | BAT2 |
| 6 | 32184384 | 32185784 | 1401 | 0.0366 | NOTCH4 |
| 4 | 107237297 | 107238115 | 819 | 0.0373 | AIMP1;TBCK |
| 16 | 89161281 | 89166593 | 5313 | 0.0382 | ACSF3 |
| 17 | 46682248 | 46683845 | 1598 | 0.0386 | LOC404266;HOXB6 |
| 11 | 76380921 | 76381270 | 350 | 0.0389 | LRRC32 |
| 10 | 81071754 | 81077514 | 5761 | 0.0392 | ZMIZ1 |
| 17 | 74522215 | 74526633 | 4419 | 0.0392 | CYGB |
| 7 | 1988561 | 1990249 | 1689 | 0.0392 | MAD1L1 |
| 17 | 75314855 | 75316256 | 1402 | 0.0399 | SEPT9 |
| 6 | 33259148 | 33265011 | 5864 | 0.0399 | RGL2 |
| 6 | 105628000 | 105628125 | 126 | 0.0399 | POPDC3 |
| 1 | 24645286 | 24646989 | 1704 | 0.0402 | GRHL3 |
| 22 | 39711834 | 39713884 | 2051 | 0.0413 | RPL3;SNORD83A;RNU86 |
| 6 | 31615289 | 31618404 | 3116 | 0.0413 | BAT3 |
| 1 | 154941696 | 154945535 | 3840 | 0.0422 | SHC1 |
| 10 | 94607793 | 94608095 | 303 | 0.0422 | EXOC6 |
| 2 | 43903137 | 43904094 | 958 | 0.0427 | PLEKHH2;LOC728819 |
| 6 | 31777084 | 31782129 | 5046 | 0.0427 | HSPA1L;HSPA1A |
| 13 | 78493568 | 78493733 | 166 | 0.0435 | EDNRB |
| 6 | 31619858 | 31620010 | 153 | 0.0435 | BAT3 |
| 17 | 26692283 | 26696328 | 4046 | 0.0435 | SEBOX;VTN |
| 6 | 30296375 | 30298739 | 2365 | 0.0435 | TRIM39 |
| 6 | 31869467 | 31869633 | 167 | 0.0436 | ZBTB12 |
| 6 | 163832900 | 163835415 | 2516 | 0.0436 | QKI |
| 5 | 137548253 | 137549142 | 890 | 0.0447 | CDC23 |
| 6 | 32791332 | 32798954 | 7623 | 0.0447 | TAP2 |
| 6 | 33172988 | 33173282 | 295 | 0.0447 | HSD17B8 |
| 1 | 32715398 | 32718605 | 3208 | 0.0455 | LCK |
| 6 | 28303534 | 28304675 | 1142 | 0.0461 | ZNF323 |
| 6 | 30594417 | 30595399 | 983 | 0.0461 | C6orf134 |
| 6 | 30566375 | 30571233 | 4859 | 0.0475 | PPP1R10 |
| 10 | 106093608 | 106097091 | 3484 | 0.0479 | ITPRIP |
| 6 | 30157290 | 30160369 | 3080 | 0.0500 | TRIM26 |
